# Subgaleal and Epidural Hematomas Secondary to Calvarial Infarcts: An Uncommon Presentation of Sickle Cell Disease

**DOI:** 10.7759/cureus.57961

**Published:** 2024-04-10

**Authors:** Marina Shenouda, Praneet S Paidisetty, Phuong D Nguyen

**Affiliations:** 1 Ophthalmology, University of Texas Health Science Center at Houston McGovern Medical School, Houston, USA; 2 Plastic Surgery, University of Texas Health Science Center at Houston McGovern Medical School, Houston, USA; 3 Division of Plastic & Reconstructive Surgery, Department of Surgery, University of Colorado Anschutz Medical Campus, Aurora, USA

**Keywords:** skull bone infarction, craniofacial surgery, epidural hematoma, subgaleal hematoma, calvarial infarct, sickle cell disease

## Abstract

We present the case of a 13-year-old male with sickle cell disease (SCD) who presented to the emergency department with a severe headache secondary to calvarial infarcts with associated epidural and subgaleal hematomas. This case was complicated by external compression of the superior sagittal sinus by the hematomas as seen on magnetic resonance imaging. Management included supportive treatment of pain and swelling. This case emphasizes skull infarctions with associated hematomas as a possible differential diagnosis for patients with SCD presenting with headaches and scalp swellings.

## Introduction

Sickle cell disease (SCD) is a hereditary hemoglobinopathy common among individuals from Africa, the Mediterranean, India, and the Arabian peninsula. It is characterized by mutations in the hemoglobin β chain, leading to occlusive sickling of red blood cells when exposed to hypoxia. Common physiological manifestations include constitutional symptoms, anemia, functional asplenia, and end-organ damage due to micro- and macrovascular infarcts [1]. These infarcts may occur in the bone, leading to pain, swelling, and hematomas [2]. Infarcts secondary to SCD most commonly occur in the long bones and axial skeleton. Much less common are infarcts in the skull, which lead to localized bone marrow edema with associated swelling. In cases of extensive bone infarction, associated hematomas may be present. These infarcts may present as headache, swelling, and/or facial pain [3]. We present a patient with skull infarcts leading to epidural and subgaleal hematomas, representing an unusual manifestation of sickling disorders.

## Case presentation

A 13-year-old African American boy with a past medical history of SCD presented to the Memorial Hermann emergency department (ED) in distress due to severe holocranial headache and chest pain. He reported having recent headaches of similar severity and onset as well as dyspnea secondary to chest pain. He also reported a one-week history of a mild dry cough. A review of the systems was positive for fever and negative for rash, weight loss, abdominal pain, and vomiting. Chest X-ray ruled out acute chest syndrome.

The patient had several past hospitalizations for vaso-occlusive crises in the chest, head, and bilateral hips. Of note, the patient was previously hospitalized in 2011 for a splenectomy, in 2015 for painless periorbital infarction, and in 2021 for a subgaleal hematoma. He had a right subclavian port-a-cath placement in 2021. His home medications include hydroxyurea, prophylactic penicillin, and monthly crizanlizumab infusions. Preliminary assessment in the ED was concerning for a sickle cell crisis, and the patient was admitted to the Memorial Hermann Children’s Hospital. Upon chart review, during a prior admission two years ago, computed tomography (CT) of the brain without contrast showed extra-axial fluid collections, consistent with subgaleal hematomas. They were found to have completely resolved on repeat imaging.

**Figure 1:**
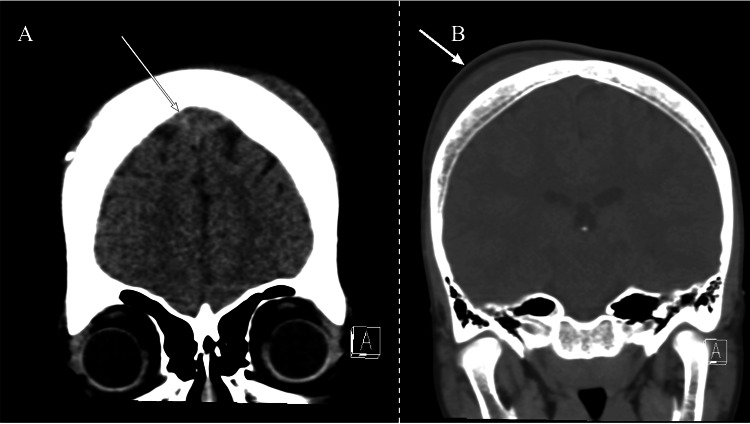
Coronal CT of epidural and subgaleal hematomas. Epidural (A) and subgaleal (B) hematomas are indicated by arrows.

**Figure 2:**
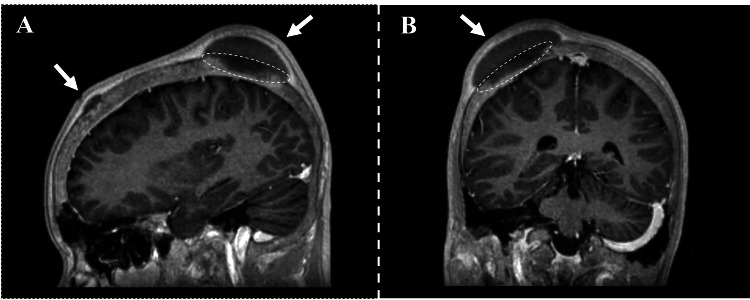
Sagittal (A) and coronal (B) MRI with contrast. Hypointense signals of the right parietal and anterior frontal calvarium suggest bony infarction. The bone infarctions are indicated with circles, and the adjacent hematomas are indicated with arrows.

**Figure 3:**
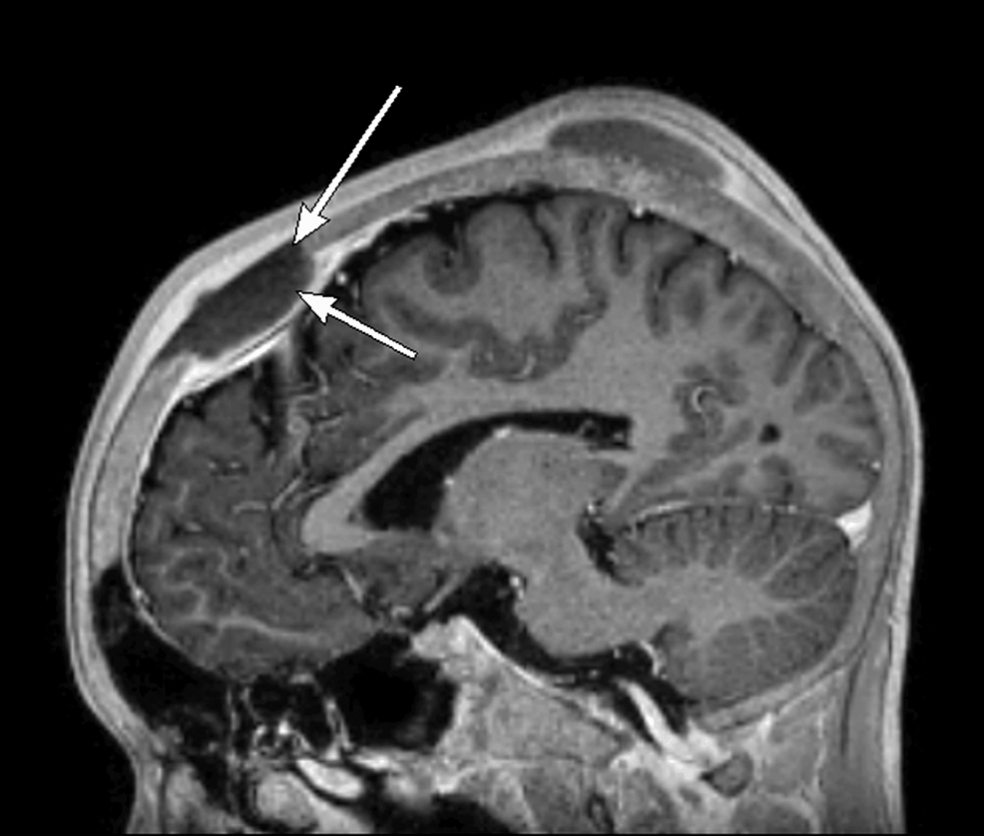
Sagittal MRI with contrast. Post-contrast MRI shows compression and inferior displacement of the superior sagittal sinus secondary to the overlying epidural hematoma. Arrows indicate bone infarction (top arrow) and associated hematoma (bottom arrow).

Given his history of subgaleal hematomas, neurology was consulted for a workup of the acute headache. On external cranial examination, a 2-3 cm area of localized, tender swelling over the right parietal region was identified. Two smaller areas of tenderness and swelling over the left occipital regions were also present. On CT of the brain, an anterior frontal epidural fluid collection and bilateral frontal subgaleal collections most consistent with subacute hematomas were visualized (Figure 1). Magnetic resonance imaging (MRI) of the brain showed hematomas in continuity with focal bone lesions (Figure 2). The epidural hematoma was seen to cause compression of the anterior third of the superior sagittal sinus with inferior displacement on imaging (Figure 3). Magnetic resonance venography (MRV) of the brain demonstrated patency of the superior sagittal sinus despite compression secondary to the epidural hematoma.

Craniofacial surgery was consulted and recommended compression of the hematomas with a compressive elastic bandage or swim cap. Following the implementation of this intervention, the patient reported a reduction in scalp swellings, though his headache persisted. He had been receiving opioids for his chest pain, and ondansetron and diphenhydramine were added for the management of his headache given this regimen’s prior success at this hospital in patients with persistent migraines. Surgical intervention was not recommended at the time due to stable non-expanding hematomas without occlusion of vascular structures. The patient was subsequently discharged two weeks later upon resolution of his headache and chest pain, which was presumed to be secondary to an acute vaso-occlusive crisis and managed by the primary pediatrics team. He was recommended to follow up with pediatric neurology on an outpatient basis.

## Discussion

In SCD, red blood cells have abnormally structured hemoglobin S molecules, causing them to sickle in conditions of cellular stress. Abnormal red blood cell morphology causes the cells to clump in vessels, leading to vaso-occlusion and the painful “sickle cell crises” associated with the disease [1]. Skull bone infarcts are a rare occurrence in those with SCD and co-occurrence with epidural hematomas is even more uncommon [2]. Infarction of the bone is generally due to occlusion of microvasculature from a variety of causes, including mechanical obstruction, thrombosis, or gas/fat emboli, ultimately leading to osteonecrosis [4-6]. In general, pediatric calvarial infarctions are highly likely to be due to SCD or other sickling disorders; related case reports describe this pathology in the context of SCD [7-13].

Our case documents a presentation of SCD that is rarely reported in the literature. Although subgaleal and epidural hematomas have been individually described in previous case reports in patients with SCD and other comorbid genetic disorders such as glucose-6-phosphate dehydrogenase deficiency, the presence of both is extremely uncommon in isolated SCD [8-11]. In these cases, hematomas tended to be associated with an adjacent bone infarction seen on imaging. These cases often present with a headache, though more serious causes for the headache should be ruled out. Only one prior case has described both subgaleal and epidural hematomas occurring concurrently in the same patient with isolated SCD, but external compression of the superior sagittal sinus was not seen unlike in our case [12]. In the case of focal signs or compression of neurological structures, surgical intervention is indicated. Komarla et al. reported a case in which a patient with SCD presented with headache and left-sided weakness [13]. The patient was found to have a large right-sided epidural hematoma on MRI and signal intensities consistent with a calvarial bone infarction. The acute neurological deficits were likely due to a mass effect from the overlying epidural hematoma. The patient underwent emergent evacuation of the hematoma. As the overlying calvaria was not noted to be abnormal, cranioplasty was not indicated [13].

Headaches are a common finding in children with SCD, and their management is often challenging as the exact etiology may be difficult to identify. Serious causes of headaches in patients with SCD include cerebral infarction and cerebral vasculopathy which should be ruled out [14]. In this case, an acute tension headache was briefly considered as the cause for the patient’s distress due to the diffuse nature of the pain, though the patient’s acute distress made this less likely. Although bilateral migraines are more common in children, an acute migraine was deemed unlikely due to a lack of aura and photophobia. Infectious processes were also unlikely due to the absence of fever, normal white blood cell count, and a negative chest X-ray. Given the patient’s previous periorbital infarctions and epidural and bilateral subgaleal hematomas, a recurrence of his calvarial infarcts seemed the most likely cause of his headache. This was confirmed on imaging, and MRV further ruled out venous sinus thrombosis as the cause of the headache. The formation of the scalp swellings once the patient had arrived at the ED may have been due to the development of the hematomas due to the altered nearby bony and periosteal structures secondary to infarction [15].

In accordance with the literature [7-13], we recommend that patients be managed conservatively to minimize hematoma formation and expansion. Adequate pain control is a cornerstone in the management of such patients. We recommend a multimodal pain regimen to provide sufficient analgesia. In the case of expansion of hematomas resulting in symptomatic occlusion of cranial vasculature, neurosurgery and craniofacial surgery should be consulted for possible intervention. Patients should undergo serial neurological examinations to monitor for the development of any focal neurological deficits, which may indicate occlusion of neurovascular structures secondary to mass effect. In our case, although our patient had compression of the superior sagittal sinus, MRV confirmed its patency and he had no focal neurological signs and was therefore managed conservatively. In the case of focal signs or compression of neurological structures, surgical intervention is likely indicated [13]. High clinical suspicion is needed in case emergent surgical interventions such as craniectomy and/or cranioplasty are required for decompression.

## Conclusions

This case highlights an uncommon cause of headaches in patients with SCD. Sickle cell bone infarctions may occur in the calvarium, leading to the formation of associated hematomas and compression of the superior sagittal sinus and manifesting as headaches. Management for such cases is largely conservative in the absence of focal neurologic signs and consists of reducing swelling with gentle compression and pain control. However, primary teams should be aware of when to consult for high-level surgical management such as craniectomy in the case of extensive bone infarcts or symptomatic compression of neurologic vasculature.
